# Precise MEP monitoring with a reduced interval is safe and useful for detecting permissive duration for temporary clipping

**DOI:** 10.1038/s41598-020-60377-9

**Published:** 2020-02-26

**Authors:** Masahiro Kameda, Tomohito Hishikawa, Masafumi Hiramatsu, Takao Yasuhara, Kazuhiko Kurozumi, Isao Date

**Affiliations:** Department of Neurological Surgery, Okayama University Graduate School of Medicine, Dentistry and Pharmaceutical Sciences, 2-5-1 Shikata-cho, Kita-ku, Okayama-Shi, Okayama, 700-8558 Japan

**Keywords:** Stroke, Stroke

## Abstract

Although temporary clipping of the parent artery is an indispensable technique in clipping surgery for intracranial aneurysms, the permissive duration of temporary clipping is still not well known. The aim of this study is to confirm the safety of precise motor evoked potential (MEP) monitoring and to estimate the permissive duration of temporary clipping for middle cerebral artery (MCA) aneurysm based on precise MEP monitoring results. Under precise MEP monitoring via direct cortical stimulation every 30 seconds to 1 minute, surgeons released a temporary clip and waited for MEP amplitude to recover following severe (>50%) reduction of MEP amplitude during temporary clipping. Precise MEP monitoring was safely performed. Twenty-eight instances of temporary clipping were performed in 42 MCA aneurysm clipping surgeries. Because precise MEP monitoring could be used to determine when to release a temporary clip even with a severe reduction in MEP amplitude due to lengthy temporary clipping, no patients experienced permanent postoperative hemiparesis. Based on logistic regression analysis, if a temporary clip is applied for 312 seconds or more, there is a higher probability of a severe reduction in MEP amplitude. We should therefore release temporary clips after 5 minutes in order to avoid permanent postoperative hemiparesis.

## Introduction

Temporary clipping in aneurysm surgery results in decreased aneurysmal pressure, which enables neurosurgeons to prevent premature rupture and facilitates the exposure of the intracranial aneurysm. In spite of these advantages, lengthy temporary clipping can cause ischemic damage. To date, the permissive duration of temporary clipping free of postoperative neurological deficits, especially that based on electrophysiological monitoring, remains to be fully elucidated.

As an intraoperative electrophysiological monitoring technique, motor evoked potential (MEP) has become an essential component of recent aneurysm surgery because the sensitivity of MEP monitoring for postoperative motor deficits is higher than that of sensory evoked potential (SEP) monitoring^1–6^. MEP monitoring via direct cortical stimulation (DCS) can be more reliably performed on an immobile subject even during microscopic procedures, compared with that via transcranial electrical stimulation (TES)^3,7^.

Based on this background, we hypothesized that precise MEP monitoring via DCS at a reduced interval (every 30 seconds to 1 minute) could be used to determine the appropriate timing for releasing a temporary clip without permanent postoperative hemiparesis, even in cases involving lengthy temporary clipping. We also statistically estimated the permissive duration of temporary clipping by analyzing instances of temporary clipping during MCA aneurysm surgeries.

## Results

### Precise MEP can be performed safely

We performed precise MEP monitoring via DCS in MCA (n = 47) and IC (n = 30) aneurysms that could be safely performed without inducing body motion, which interrupts the microdissection. No complications such as subdural hematoma were induced by precise MEP monitoring.

### Precise MEP monitoring could be used to determine the appropriate timing to release a temporary clip in MCA aneurysm clipping surgeries and prevent the occurrence of permanent postoperative hemiparesis

After applying a temporary clip to the MCA M1 segment, we monitored the MEP amplitude via frequent DCS every 30 seconds to 1 minute. We released a temporary clip if a severe (>50%) reduction of MEP amplitude occurred during temporary clipping. If we needed to apply a temporary clip two or more times, after confirming the recovery of the MEP amplitude, we reapplied a temporary clip. Even if we experienced a severe reduction in MEP amplitude during temporary clipping, we could confirm the recovery of the amplitude after releasing the clip. We could subsequently use another temporary clip and ultimately apply a permanent clip for the MCA aneurysm. Although two patients showed minor weakness on the contralateral side immediately after recovery from anesthesia, neither exhibited weakness the following day. As a result, no patient experienced permanent postoperative hemiparesis at the time of discharge.

### Five minutes is the permissive duration for temporary clipping in MCA aneurysm surgery

**Figure 1:**
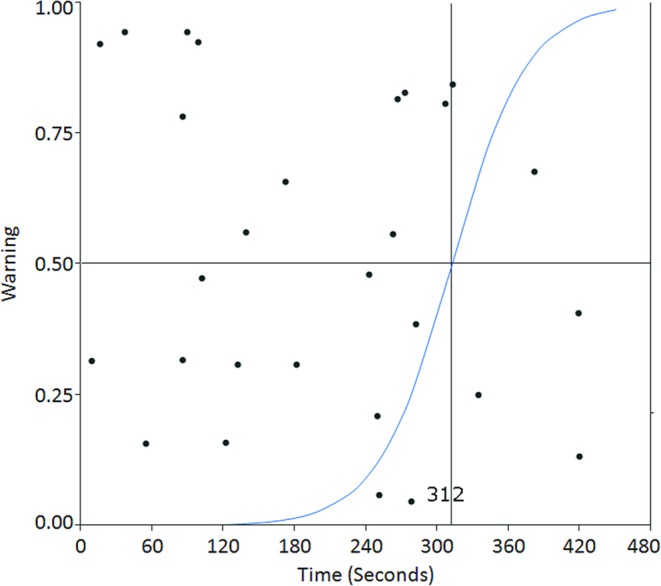
Based on logistic regression analysis based on the 28 instances of temporary clipping, if a temporary clip needed to be applied for 312 seconds or more, there was a higher probability (R^2^: 0.55) of encountering a severe reduction in the MEP amplitude.

Severe reduction in MEP amplitude was confirmed six times. The rate of change in MEP amplitude in these cases was 15.7 ± 4.3% (average ± standard error) of initial amplitude (i.e., average 84.3% reduction in MEP amplitude). In these cases, the change in MEP amplitude was discovered 348 ± 29 seconds after applying a temporary clip to the MCA M1 segment. When an alert for severe reduction in MEP amplitude was issued, surgeons released the temporary clip 366 ± 34 seconds after applying it. In contrast, severe reduction in MEP amplitude did not occur 22 times. In these cases, surgeons released the temporary clip 176 ± 21 seconds after applying it to the MCA M1 segment. Based on a logistic regression analysis, if a temporary clip needs to be applied for 312 seconds or more, there is a higher probability of causing a severe reduction in the MEP amplitude (Fig. 1). From the ROC curves, the optimum cutoff time to induce severe reduction in the MEP amplitude is 251 seconds or more (AUC 0.93, sensitivity 1, specificity 0.73, Youden index 0.73).

We also evaluated whether multiple temporary clipping events affect the permissive duration of temporary clipping. From a logistic regression analysis using the data of the first temporary clipping (n = 9), if a temporary clip needs to be applied for 297 seconds or more, there is a higher probability of causing a severe reduction in the MEP amplitude. From the ROC curves, the optimum cutoff time to induce a severe reduction in the MEP amplitude was found to be 278 seconds or more (AUC 0.93, sensitivity 1, specificity 0.86, Youden index 0.86). In contrast, when we performed a logistic regression analysis using the data of the second and subsequent temporary clippings (n = 19), if a temporary clip needs to be applied for 323 seconds or more, there is a higher probability of generating a severe reduction in the MEP amplitude. From the ROC curves, the optimum cutoff time to induce a severe reduction in the MEP amplitude was 251 seconds or more (AUC 0.93, sensitivity 1, specificity 0.73) 382 seconds or more (AUC 0.93, sensitivity 0.75, specificity 1, Youden index 0.75). (Table 1)

**Table 1:** Statistical estimation for permissive time of temporary clipping usage based on the results for all temporary clippings, those for the first temporary clipping only, and those for the second and subsequent temporary clippings.

	All temporary clippings	1st temporary clipping only	2nd and subsequent temporary clippings
Sensitivity 1	251 seconds (specificity 0.73)	278 seconds (specificity 0.86)	251 seconds (specificity 0.73)
Specificity 1	335 seconds (sensitivity 0.67)	335 seconds (sensitivity 0.5)	382 seconds (sensitivity 0.75)
Cutoff time from the ROC curve by the Youden index	251 seconds (*1)	278 seconds (*2)	382 seconds (*3)
Probability >50% from logistic regression analysis	312 seconds	297 seconds	323 seconds

(*1: sensitivity 1, specificity 0.73, Youden index 0.73).

(*2: sensitivity 1, specificity 0.86, Youden index 0.86).

(*3: sensitivity 0.75, specificity 1, Youden index 0.75).

As we mentioned previously, two patients showed minor weakness on the contralateral side immediately after recovery from anesthesia, but neither exhibited weakness the following day. The overall temporary clipping times were 703 ± 563 and 668 ± 182 seconds for the patients with transient postoperative weakness (n = 2) and without transient postoperative weakness (n = 7), p = 0.88, respectively. The overall temporary clipping time did not play any role in predicting postoperative weakness in this series.

As we showed above, precise MEP monitoring via DCS is beneficial because we could determine the appropriate timing at which to release a temporary clip, and no patients experienced permanent postoperative hemiparesis. Moreover, based on the statistical analysis for precise MEP monitoring results, regardless of how many times temporary clippings are employed, surgeons should prepare for the release of a temporary clip after 5 minutes to avoid permanent postoperative hemiparesis.

### Representative case: Case #1

**Figure 2:**
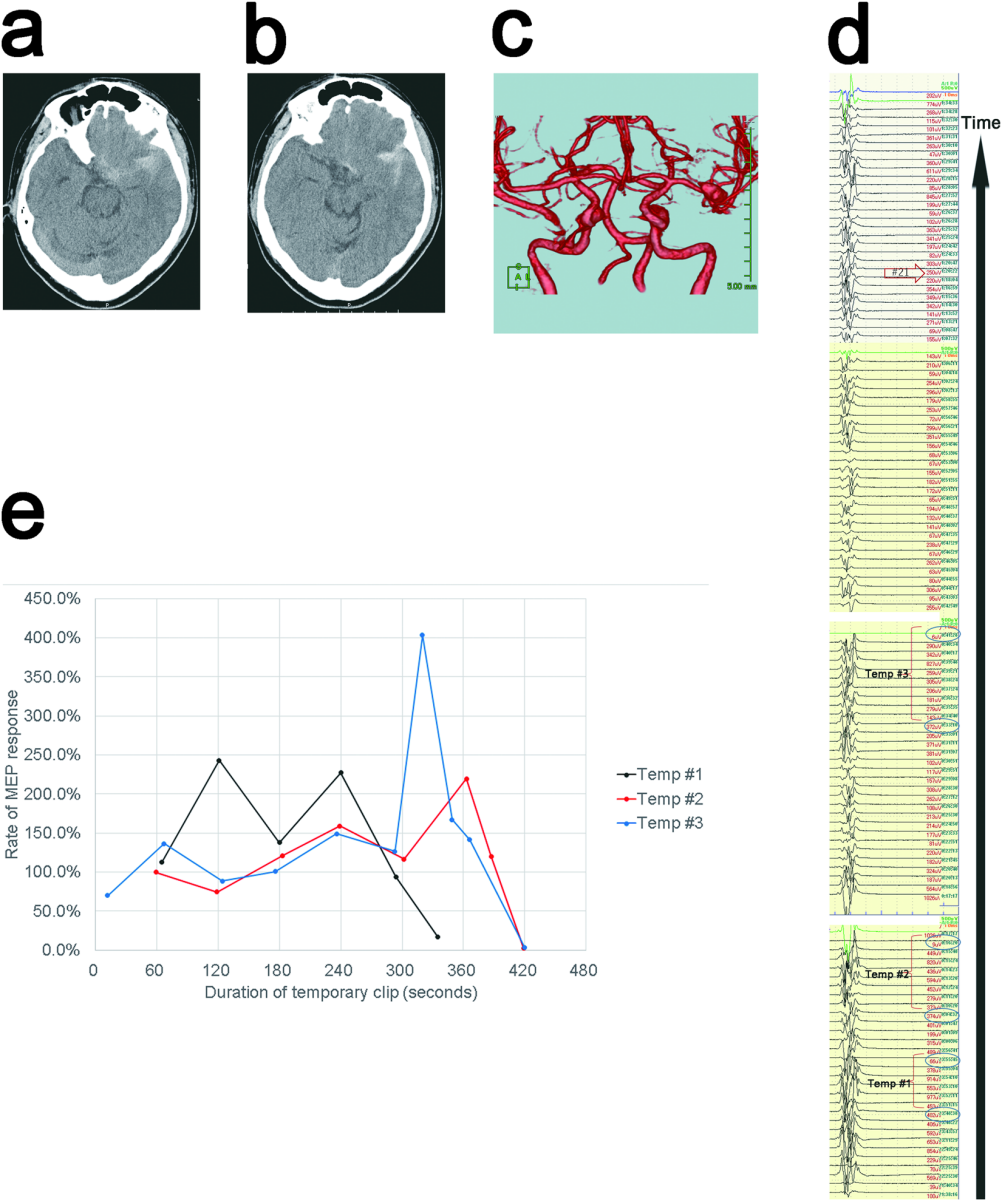
A CT scan taken at admission showed SAH (Fisher group 3) (Fig. 2a,b), and 3DCTA showed an intracranial aneurysm in the left MCA bifurcation (Fig. 2c). Intraoperative monitoring results of precise MEP via DCS with reduced interval shows three instances of temporary clipping (Temp #1, Temp #2, and Temp #3) with severe reduction in the MEP amplitude. After applying a permanent clip (Sugita #21) to the aneurysm, the MEP amplitude was preserved (Fig. 2d). The graph shows three instances of MEP amplitude after applying a temporary clip. It is notable that the MEP amplitude showed an extreme increase (>400%) before showing a severe reduction, as seen in Temp #3 (Fig. 2e).

A 40-year-old male patient of Hunt-Kosnik grade I underwent aneurysm surgery for a ruptured intracranial aneurysm in the left MCA bifurcation (Fig. 2a–c). This procedure was performed as an emergency operation. We monitored the MEP amplitude approximately every minute via DCS after initiating the microdissection of the area surrounding the aneurysm. Appling a temporary clip to the MCA M1 segment induced a severe reduction in the MEP amplitude three times (reduction rates: 83.6%, 97.6%, and 97.1% for the first, second, and third time, respectively). At the time of the severe reduction in the MEP amplitude, the surgeons released the temporary clip, waited for the recovery of the MEP amplitude, and then reapplied the clip. After we confirmed the recovery of the MEP amplitude after three instances of temporary clipping, we finally applied a No 21 Sugita clip (Mizuho Co., Ltd., Tokyo, Japan) to the aneurysm. After clipping the aneurysm, we continued MEP monitoring via DCS for approximately 15 minutes and confirmed the restoration of the MEP amplitude. We then removed the strip electrode for DCS (Fig. 2d). It is notable that the MEP amplitude showed an extreme increase (>400%) before showing a severe reduction, as seen in Temp #3 (Fig. 2d,e). Although the patient showed minor weakness on the contralateral side immediately after recovery from anesthesia, he exhibited no weakness the following day, and he experienced no permanent postoperative motor deficit.

## Discussion

In this study, we could safely perform precise MEP monitoring, and precise MEP monitoring via DCS with a reduced interval is useful because we could determine the appropriate timing at which to release a temporary clip. Additionally, we found that surgeons should release a temporary clip of the MCA M1 segment after a lapse of 5 minutes in order to avoid permanent motor deficits.

We consider precise MEP monitoring to be especially useful in cases for which lengthy repetitive instances of temporary clipping are required, as was shown in the representative case. Because we monitored the MEP amplitude approximately every minute via DCS, even when a severe reduction in the MEP amplitude after applying a temporary clip was observed, the surgeons could release the clip, wait for the recovery of the MEP amplitude, and then reapply the clip. In the representative case, the second and third temporary clip induced very severe reduction in MEP amplitude to less than 3% of control at 419 or 420 seconds after applying the clip, causing the monitoring assistant to issue a prompt alert to the surgeons, after which the surgeons released the temporary clip with a temporal occlusion time 466 or 440 seconds. Even in such lengthy repetitive times of temporary clipping, we could confirm the recovery of the MEP amplitude after releasing the clips, and a permanent clip could be applied in all instances (Fig. 2d,e, Table 2)

**Table 2:** Results of 28 temporary clippings with precise MEP monitoring.

# Case	Temporary clipping #	Duration from applying a temporary clip to delivering DCS just before releasing a temporary clip(s)	Duration from applying a temporary clip to releasing a temporary clip(s)	Rate of change in MEP amplitude (post/pre)	Alert warning due to severe reduction of MEP amplitude	Unruptured/Ruptured	Overall time of temporary clipping(s)	Transient contralateral weakness	Permanent postoperative hemiparesis
1	1	335	360	16.40%	+	ruptured	1266	+	−
	2	419	466	2.40%	+				
	3	420	440	2.90%	+				
4	1	267	300	115.60%	−	unruptured	1150	−	−
	2	263	290	67.50%	−				
	3	282	300	63.30%	−				
	4	250	260	59.10%	−				
8	1	139	185	114.70%	−	unruptured	615	−	−
	2	243	254	76.50%	−				
	3	86	102	123.00%	−				
	4	55	74	64.20%	−				
12	1	90	127	103.90%	−	unruptured	359	−	−
	2	86	92	52.00%	−				
	3	16	24	78.30%	−				
	4	102	116	90.70%	−				
19	1	278	285	24.20%	+	unruptured	1428	−	−
	2	313	320	119.80%	−				
	3	251	257	24.60%	+				
	4	382	389	23.90%	+				
	5	172	177	68.20%	−				
24	1	132	140	198.20%	−	unruptured	140	+	−
29	1	307	322	223.50%	−	unruptured	755	−	−
	2	273	282	145.50%	−				
	3	122	151	520.00%	−				
33	1	182	182	70.20%	−	unruptured	182	−	−
57	1	9	30	98.30%	−	unruptured	184	−	−
	2	37	55	96.40%	−				
	3	99	99	99.30%	−				

In this study, two patients showed transient contralateral weakness postoperatively (cases #1 and #24, Table 2). For patient #1 with a short M1, we fortunately confirmed the recovery of MEP amplitude after releasing a temporary clip, although we employed lengthy temporary clips several times. For patient #24 without a short M1, there was no alert warning because there was no severe reduction of MEP amplitude; nevertheless, the patient did show transient contralateral weakness postoperatively. Based on these cases, there seems to be no relationship between a short M1, the permissive duration of a temporary clip, and postoperative transient weakness. However, a lentriculostriate artery originating from the M2 segment in the MCA could affect the permissive duration of a temporary clip, especially in cases with a short M1. In such cases, precise MEP monitoring would be beneficial for detecting the appropriate timing for releasing temporary clips.

The permissive duration of temporary clipping for an MCA bifurcation aneurysm would be affected by the placement of the temporary clip in terms of whether it is proximal or distal to the lentriculostriate artery on the M1 segment in the MCA. In our case, we consistently applied a temporary clip distal to the lentriculostriate artery on the M1 segment in the MCA. The magnitude of leptomeningeal anastomosis would also affect the permissive duration of temporary clipping. Unfortunately, it is difficult to evaluate this point before aneurysm surgery, especially in emergency cases involving ruptured aneurysms. Previous reports showed that temporary clipping of up to 10 minutes could be safely achieved^8^. In contrast, our study showed that temporary clipping of more than 5 minutes carried a higher probability of a severe reduction in MEP amplitude, which could induce permanent postoperative hemiparesis. This discrepancy is due to the difference in study design. In the previous study, they evaluated the duration of temporary clipping and postoperative ischemic lesions shown in postoperative MR or CT and neurological deficits. In our study, however, we performed aneurysm surgery with precise MEP monitoring with reduced intervals, and surgeons could quickly release the temporary clip if a severe reduction in MEP amplitude occurred. MEP amplitude could subsequently be recovered, and patients did not end up with permanent motor deficits. Taken together, temporary clipping for more than 5 minutes triggered ischemic penumbra, while temporary clipping for more than 10 minutes triggered ischemic core.

Another report indicated that the safe duration for temporary clipping of an MCA aneurysm was 2.4 min^9^. This safe duration was also statistically analyzed based on the results of MEP monitoring, but 2.4 min is significantly shorter than our results. This discrepancy is due to the difference in MEP monitoring procedures. We analyzed the results obtained from precise MEP monitoring with reduced intervals, whereas they analyzed the MEP amplitude before and after temporary clipping. Then, they recognized the duration of temporary clipping in patients without MEP changes as the safe duration. That is why the safe duration in patients without MEP changes could be longer than the measured values, as they made mention of in their manuscript.

In this study, we also evaluated how multiple temporary clippings affect the permissive duration of temporary clipping usage. The permissive time for second and subsequent temporary clippings (n = 19) tended to be longer than that for the first temporary clipping (n = 9). However, when taken together with the results from a logistic regression analysis and an ROC analysis (Table 2), either in the first temporary clipping or in the second and subsequent temporary clipping, we should prepare for the release of a temporary clip after 5 minutes in order to prevent permanent motor deficits. Moreover, we also evaluated whether the overall time of temporary clipping usage predicts MEP changes or postoperative weakness. The overall time of temporary clipping usage was not predictive of either because we were able to determine the appropriate timing to release the temporary clipping and surgeons could quickly release temporary clippings within the ischemic penumbra even if a severe reduction in MEP amplitude occurred.

Regarding the permissive duration of temporary clipping in MCA aneurysm surgeries, 5 minutes is the optimum cutoff time and applying a temporary clip for more than 5 minutes induces a higher probability of a severe reduction in the MEP amplitude, which can also be applied to aneurysm surgeries with MEP monitoring via TES only, as well as to aneurysm surgeries without MEP monitoring. MEP monitoring cannot always be performed, especially in emergency surgeries for ruptured aneurysms. Even if MEP monitoring cannot be performed in MCA aneurysm surgeries, we should prepare for the release of a temporary clip after 5 minutes, thereby enabling us to prevent permanent motor deficits. However, we are aware that surgeons sometimes require a lengthy temporary clipping (>5 minutes) for microdissection, especially in SAH cases. In such cases, some sort of brain protection (e.g., radical scavenger) should be considered for reducing the risk of postoperative hemiparesis.

As we mentioned above, our study showed that temporary clipping of more than 5 minutes carried a higher probability of a severe reduction in MEP amplitude. However, in a strict sense, the duration of safe temporary clipping might be a little bit longer. We statistically analyzed 28 instances of temporary clipping that were performed in 9 of 42 patients with MCA aneurysms, and 8 of 9 patients underwent surgery for unruptured aneurysms, which is why surgeons needed to perform clipping without causing postoperative motor deficits. Because of this, the surgeons did not employ lengthy temporary clipping, and 22 instances (22/28 = 79%) of temporary clipping in this study were completed within 5 minutes. In spite of this limitation of our study, our finding that 5 minutes is the optimum cutoff time can be applied to aneurysm surgery with TES alone, as well as to aneurysm surgeries without MEP monitoring.

## Methods

### Patients

In this study, we reviewed our 110 consecutive aneurysm surgeries for intracranial aneurysms. The three major locations of the aneurysms were the MCA, IC, and Acom, and the numbers of aneurysms in each location were 47, 30, and 28, respectively. Precise MEP monitoring via DCS was mainly performed for MCA and IC aneurysms. These surgeries were performed from 2012 to 2016.

### Ethical approval

All procedures performed in studies involving human participants were in accordance with the ethical standards of the Okayama University Graduate School of Medicine ethical committee and with the 1964 Helsinki declaration and its later amendments or comparable ethical standards. This study was approved by the Ethics Committee of the Okayama University Graduate School of Medicine, Dentistry and Pharmaceutical Sciences and Okayama University Hospital (#1711-031). Because this is a retrospective study, the authors received ethical approval for the use of an opt-out methodology.

### Anesthesia

A muscle relaxant is administrated in the induction of anesthesia. Once anesthesia was induced, no additional muscle relaxant was administrated throughout the MEP monitoring, and patients were effectively maintained under the anesthetic management by remifentanil hydrochloride and an inhalation analgesic (sevoflurane or desflurane) or remifentanil hydrochloride and a continuous intravenous injection of propofol throughout the operations. If we were unable to identify an MEP response via TES, the muscle relaxant was reversed by sugammadex sodium. After confirming the MEP response via TES, we began the aneurysm surgery.

### MEP monitoring

TES was performed by C3-C4 stimulation and recorded at the bilateral thenar eminences. Stimulating intensity was set at 20% above the minimum intensity to obtain an MEP response via TES. After craniotomy and the opening of the dura, DCS was performed by inserting a strip electrode with 20 electrodes in the subdural space to the motor cortex for the hand. MEP response via DCS was recorded at the contralateral thenar eminence. Each electrode in the strip electrode was stimulated, and we determined the best electrode for MEP monitoring by checking the MEP amplitude. By confirming the minimum intensity to obtain an MEP response on the best electrode, the stimulating intensity during MEP monitoring was set at 20% above this minimum intensity to obtain an MEP response. TES and DCS were performed using a 5-train stimulation (pulse width, 500 µs).

### Precise MEP monitoring via DCS

After initiating microdissection near the intracranial aneurysm, precise MEP monitoring was performed via DCS every 30 seconds to 1 minute. Based on previous reports^3,9–11^, we regarded a >50% reduction of MEP amplitude compared with control as a severe reduction. If a severe reduction of MEP amplitude compared with control did not occur, the surgeons continued the microdissection and prepared for the safe application of a permanent clip. If, however, a severe reduction of MEP amplitude occurred during temporary clipping, an alert was issued, and the surgeons released the temporary clip and waited for the MEP amplitude to recover. After confirming the recovery, the surgeons resumed the microdissection around the intracranial aneurysm. After applying a permanent clip, MEP monitoring was continued for at least 10 minutes. If a stable MEP amplitude could not be obtained after applying the permanent clip, the surgeons released the clip and reapplied it in order to preserve the blood flow of the parent artery. After confirming a stable MEP amplitude via DCS and TES, the strip electrode was removed and the dura was closed. For each surgery, a monitoring record and the data regarding time of applying a temporary clip, time of releasing a temporary clip, time of applying a permanent clip, and special notes (e.g., complications) were obtained and reviewed retrospectively.

### Statistical analysis for permissive duration of temporary clipping for MCA aneurysm surgery based on the results of precise MEP monitoring

For clipping surgeries involving precise MEP monitoring, when we statistically estimated the permissive duration of temporary clipping, that for an MCA aneurysm was not affected by the collateral blood flow via Acom and Pcom, compared with that for an IC aneurysm and Acom aneurysm, which is why instances of temporary clipping for MCA aneurysms were analyzed to estimate the permissive duration of temporary clipping in this study.

Of 47 MCA aneurysm patients, five were excluded because they underwent clipping surgeries with an STA-MCA bypass or clipping surgeries with neuroprotective measures. As a result, 42 patients underwent aneurysm surgery under anesthetic management via remifentanil hydrochloride and an inhalation analgesic (sevoflurane or desflurane) or a continuous intravenous injection of propofol. Even if a severe reduction of MEP amplitude occurred during temporary clipping, the blood pressure was maintained at the same level throughout microscopic manipulation. Neuroprotective measures (e.g., burst suppression and mild hypothermia) were not performed in these 42 patients. Because the difference between these two types of anesthetic management (i.e., inhalation analgesic or continuous intravenous injection of propofol) did not affect the stability of MEP monitoring, these 42 patients were included for statistical analysis.

**Figure 3:**
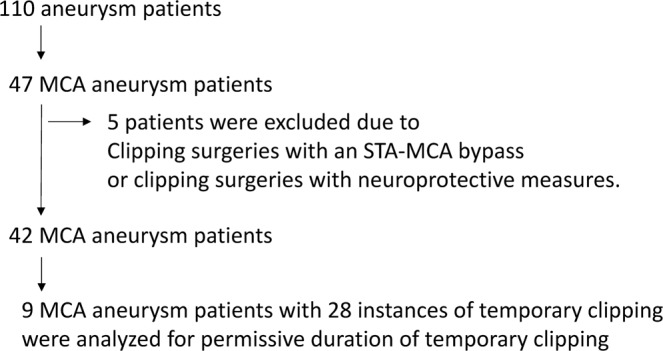
Flow diagram.

Nine of these 42 patients underwent MCA aneurysm surgery with temporary clipping of the MCA M1 segment one or more times under precise MEP monitoring via DCS. There were 28 instances of temporary clipping in 9 MCA aneurysm surgeries (1–5 instances of temporary clipping per surgery), and the resulting data were retrospectively analyzed to estimate the permissive duration of temporary clipping. (Table 2, Fig. 3).

**Figure 4:**
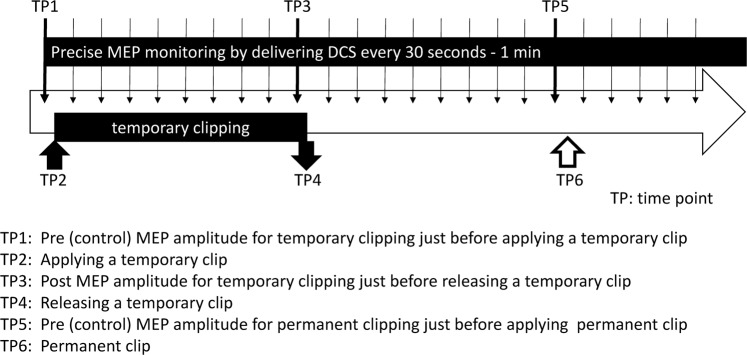
Time course for clipping surgery with temporary clipping under precise MEP monitoring.

In order to estimate the permissive duration of temporary clipping for an MCA aneurysm, we retrospectively analyzed the MEP amplitude at the following two moments: (1) just before applying a temporary clip (TP1 in Fig. 4) and (2) just before releasing a temporary clip (TP3 in Fig. 4). We also analyzed the time of the following three events: (1) applying a temporary clip (TP2 in Fig. 4), (2) delivering DCS just before releasing a temporary clip (TP3 in Fig. 4), and (3) releasing a temporary clip (TP4 in Fig. 4). The rate of change in MEP amplitude was defined as the following: (post MEP amplitude just before releasing a temporary clip [TP3 in Fig. 4])/(pre MEP amplitude just before applying a temporary clip [TP1 in Fig. 4])

The duration from applying a temporary clip (TP2 in Fig. 4) to delivering DCS just before releasing a temporary clip (TP3 in Fig. 4) and the duration from applying a temporary clip (TP2 in Fig. 4) to releasing a temporary clip (TP4 in Fig. 4) were also calculated. The permissive duration of temporary clipping was then statistically estimated. In detail, based on the previous reports^3,9–11^, if a more than 50% severe reduction in MEP amplitude compared with control occurred, an alert was issued to the surgeons. If a less than 50%, reduction in MEP amplitude compared with control occurred, this indicated no need for an alert because there was no severe reduction in the MEP amplitude.

A logistic regression analysis and a receiver operating characteristics (ROC) analysis were performed using the data for the duration from the start of applying a temporary clip (TP2 in Fig. 4) to the time of delivering DCS just before releasing a temporary clip (TP3 in Fig. 4) along with the data for the necessity of issuing an alert based on the rate of change in MEP amplitude. The permissive duration of temporary clipping for MCA aneurysm was estimated by logistic regression analysis with an optimal cutoff of 0.50, and by ROC analysis using the Youden index and/or sensitivity and/or specificity 1.

In order to evaluate how multiple temporary clippings affect the permissive duration of temporary clippings, we also performed a logistic regression analysis and an ROC analysis by dividing the results of the temporary clipping into the first temporary clipping (n = 9) and the second or subsequent temporary clippings (n = 19). To evaluate whether the overall time of temporary clippings predicts postoperative weakness, a Mann-Whitney U test was performed. All statistical analyses were performed using the software JMP10 (SAS Institute Japan, Tokyo, Japan).

## Data Availability

The datasets generated and analyzed during the current study are available from the corresponding author upon reasonable request.
